# Gluten-Free Bread: Influence of Sourdough and Compressed Yeast on Proofing and Baking Properties

**DOI:** 10.3390/foods5040069

**Published:** 2016-10-23

**Authors:** Carola Cappa, Mara Lucisano, Andrea Raineri, Lorenzo Fongaro, Roberto Foschino, Manuela Mariotti

**Affiliations:** 1 Department of Food, Environmental and Nutritional Sciences, Università degli Studi di Milano, via G. Celoria 2, 20133 Milan, Italy; mara.lucisano@unimi.it (M.L.); a.raineri@panettonevergani.it (A.R.); roberto.foschino@unimi.it (R.F.); manuela.mariotti@unimi.it (M.M.); 2 European Commission, Joint Research Centre, Directorate for Nuclear Safety and Security, P.O. Box 23 40, 76125 Karlsruhe, Germany; Lorenzo.Fongaro@ec.europa.eu

**Keywords:** gluten-free, sourdough, compressed yeast, dough, rheology, leavening, bread, texture, breadmaking, Image Analysis

## Abstract

The use of sourdough is the oldest biotechnological process to leaven baked goods, and it represents a suitable technology to improve traditional bread texture, aroma, and shelf life. A limited number of studies concerning the use of sourdough in gluten-free (GF) breadmaking have been published in comparison to those on traditional bread. The aim of this study was to compare the properties of GF breads obtained by using a previously in-lab developed GF-sourdough (SD), compressed yeast (CY; *Saccharomyces cerevisiae*) or their mixture (SDCY) as leavening agents; more specifically, it aims to confirm the findings of a previous studies and to further improve (both in terms of recipe and process) the features of the resulting GF breads. Dough pH and rheological properties were measured. Fresh and stored breads were characterized for weight, height, specific volume, crust and crumb color, moisture, water activity, crumb hardness, and porosity. The combination SDCY was effective in improving bread volume and softness when compared to SD only. Furthermore, SD- and SDCY-crumbs exhibited a less crumbly behavior during storage (69 h, 25 °C, 60% of relative humidity) in comparison to CY-breads. This study confirms the positive effect of SD in GF breadmaking, in particular when used in combination with CY.

## 1. Introduction

In gluten-free (GF) bread production, the lack of the viscoelastic gluten network makes the baking process challenging, and penalizes the quality of the final product in terms of nutritional, sensory, and technological quality, particularly with respect to bread volume and softness characteristics during storage. The rheological properties of a GF dough or batter that shows limited abilities of expansion and gas retention during leavening are critical [1]. Moreover, the high amount of starch in the formulation makes the product more prone to staling, in comparison to wheat-based breads, and reduces its shelf life. 

Various strategies can be used to improve the quality of GF bread by addressing different issues related to both the formulation and the technological process [2]. The most common ingredients used in GF breads are rice, corn, oat, pseudocereals and starches from different origins; they are generally part of a more complex recipe that includes emulsifiers, hydrocolloids, protein isolates of different sources, GF flours of different origin, or any combination of these [1,2,3]. These last ingredients are able to bind water, increase the viscosity of the system, and create, during mixing, non-gluten networks stabilized by inter- and intra-protein bonds able to mimic gluten properties. Both hydrocolloids and proteins play a strategic role in making the dough more workable and in improving the texture of the final product [4,5,6]. 

The increasing demand for high quality GF bread, clean labels, and natural products is raising the need for new approaches in GF breadmaking [7]. Unconventional treatments on raw materials, such as high pressure, have been recently studied [8,9,10] to delay starch retrogradation. Other strategies involving both formulation and breadmaking procedures concern the use of sourdough (SD), the oldest biotechnological process used to leaven baked goods. The sourdough is generated from spontaneously fermented dough constantly renewed under controlled conditions. The procedure takes its name from the sharp acidification that occurs in the mass, due to the microorganisms characterizing the dough that release organic acids and aminoacids during fermentation [11,12,13]. The microflora of sourdough generally consists of yeasts and homofermentative and heterofermentative lactobacilli. *Lactobacillus sanfranciscensis* is the typical and predominant bacterium [14], and it can form a mutualistic association with maltose-negative yeast species (*Candida milleri* or its sibling species *Candida humilis*) [15]. In fact, sourdough fermentation is very effective in improving the softening of traditional bread, increasing the volume [16], and retarding its staling, and the numerous benefits of its use have been demonstrated by many studies [16,17,18,19]. 

Nowadays, a growing number of works on the use of sourdough in GF breadmaking is being published [7,11,20,21,22,23,24,25,26]. Recently, a Type I GF-SD with a stable association between the lactic acid bacterium *Lactobacillus sanfranciscensis* and the yeast *Candida humilis* was tested in GF breadmaking [13], and the effectiveness of the addition of sourdough on GF bread quality has been proved in various formulations made of a wide range of flours. In the more recent works, beside rice and corn flour, authors have focused their attention on buckwheat [11,21,22,23], quinoa [21,24], and sorghum [21,25,26]. Data available up to now indicate that sourdough could represent an attractive tool to improve GF dough gas holding ability, as well as GF bread structure, flavor, and shelf life. 

This study is based on the findings of previous studies carried out by the authors aiming for the development of a Type I GF sourdough, from selected lactobacilli and yeasts isolated from a traditional wheat-based Type I sourdough [13] and for the evaluation of its breadmaking performance [27]. Specifically, it aimed to confirm the preliminary findings of those studies, while further improving (both in terms of recipe and process) the features of the resulting GF breads. The leavening agent and the amount of water to be added to the dough were the particular variables investigated. The Type I GF sourdough was used alone and in combination with compressed yeast, and the potential of the various leavening agents was established mainly through the evaluation of the leavening properties of the various doughs and of the quality of the final products, particularly in terms of loaf development, crumb alveolar structure, and softness decay over a three-day storage period. 

## 2. Materials and Methods

### 2.1. Gluten-Free Bread Recipe

The flour mix used for the GF bread production was composed as follows: 46.3% corn starch (Roquette Italia SpA, Alessandria, Italy), 46.3% rice flour (Beneo-Remy NV, Leuven-Wijgmaal, Belgium), and 7.4% isolated pea protein (F9; Cosucra, Warcoing, Belgium). The GF bread recipe also included 1.8% hydroxyl-propyl-methyl-cellulose (Food Grade Modified Cellulose, F4M; The Dow Company, Midland, MI, USA), 1.8% Psyllium fiber (Roeper GmbH, Hamburg, Germany), 0.7% emulsifiers (DIMODAN PH100 and PANODAN-DATEM 517; Danisco A/S, Copenhagen, Denmark), 7.4% extra-virgin olive oil, 2.5% sodium chloride, and 4.9% maltose (Merck KGaA, Darmstadt, Germany). Percentages are expressed for the flour mix, water and leavening agents excluded. The leavening agent and the amount of water to be added to the dough were the variables investigated. 

### 2.2. Gluten-Free Dough Characterization

*Leavening agents*. Three different leavening agents were used to complete the GF bread recipe: a Type I GF sourdough (SD), fresh compressed yeast (CY), and a mixture of the two (SDCY). The Type I GF sourdough was made of a stable association between *Lactobacillus sanfranciscensis* and *Candida humilis*. Briefly, a GF microbial inoculum was prepared mixing fresh cultures of a *L. sanfranciscensis* UMB26 strain and a *C. humilis* CHV strain with GF ingredients according to the protocol of Picozzi et al. [13]. The obtained dough was incubated at 25 °C until pH 4.0 was reached, and it was then continuously maintained with a back-slopping technique under the same controlled conditions. The ingredients used for the GF refreshments are reported in the same work [13]. The microbial population of SD was checked before use: it was composed of *L. sanfranciscensis* (mean value, 9.1 log CFU (Colony Forming Units)/g) and *C. humilis* species—(7.7 log CFU/g). These microbial counts were sufficient to ensure good leavening performances to the doughs during the baking process. Following the experimental plan adopted in the preliminary study [27], different GF doughs were produced: SD-dough (obtained by adding 20% SD to the GF bread recipe), CY-dough (obtained by adding 2% of fresh compressed yeast to the GF bread recipe), and SDCY-dough (obtained by adding 20% SD and 2% CY to the GF bread recipe). The 20% SD addition was chosen in accordance with the common practice in the traditional sourdough breadmaking [28]. CY was commercial grade (Casteggio Lieviti s.r.l., Casteggio, Italy). Analytics are reported below. If not differently expressed, they were performed in double. Baking trials were repeated once, and the coefficient of variation was less than 8% (referring to the specific bread loaf volume).

*Dough consistency (water addition)*. According to the recent literature [29,30] and to the authors’ preliminary studies [27,31], and on the basis of a subsequent research [32], water was added to the GF bread recipe up to a SD-dough consistency of 230 ± 10 BU and 180 ± 10 BU (Brabender Unit). In order to make comparisons with some authors’ preliminary findings [27], the sample containing sourdough, which is the most critical in terms of leavening properties and dough development, was here replicated at a 230 BU level consistency. These water absorption levels (WA, %) were assessed by means of a Brabender^®^ Farinograph (Brabender OHG, Duisburg, Germany; 300 g chamber, 25 °C). Powders were pre-mixed for 5 min; the remaining ingredients were added within the subsequent 2 min. Kneading was carried out for 15 min. The resulting dough was divided into aliquots to be further evaluated during proofing.

In total, four recipes (Table 1) were produced and analyzed:

- SD230 (SD, having a farinographic consistency of 230 ± 10 BU);
- SD180 (SD, having a farinographic consistency of 180 ± 10 BU);
- CY180 (CY, having a farinographic consistency of 180 ± 10 BU);
- SDCY180 (SDCY, having a farinographic consistency of 180 ± 10 BU).

*Dough leavening properties*. Dough development during leavening and CO_2_ production and retention were investigated with a Chopin Rheofermentometer F3 (Chopin; Villeneuve-La-Garenne, Cedex, France). A 300 g dough mass was leavened at 25 °C over a period of 16 h 30 min for SD230, 15 h for SD180, 3 h for CY180, and 16 h SDCY180; for those doughs containing the SD, the leavening was prolonged to reach pH = 4.0 ± 0.2. Furthermore, for all the doughs, these lengths were adequate to identify the Tx (time of dough porosity appearance; min), a rheofermentographic parameter frequently used to define the proofing length in a real breadmaking process. Only the height probe (254 g) of the instrument was placed on the dough, without adding any extra weight. The following indices were taken from the resulting curves, among the others: Hm (dough maximum height; mm), Hf (dough height at the end of the test; mm), Tx (time of dough porosity appearance; min), and Rc (gas retention coefficient; %).

*Dough pH evolution during proofing*. In parallel, in order to monitor yeasts and bacteria activities during proofing, the pH was continuously recorded on a 50 g dough aliquot using a pH-meter PHM 220 (Radiometer; A. De Mori Strumenti SpA, Milano, Italy). Recording stopped when 3.8 < pH < 4.0 was reached. 

### 2.3. Breadmaking Process

The breadmaking process was performed as reported by Mariotti [1], adopting some adjustments due to the different raw materials used in the current recipe. All of the powders (and SD when present) were pre-mixed with a Hobart N-50 mixer (Hobart Corporation, Troy, OH, USA) for 5 min at 60 rpm, 25 °C. The ingredients dispersed in water (maltose, NaCl and—when present—CY) were then added, followed by the remaining water and the extra-virgin olive oil. All the ingredients were added within the first 2 min of mixing, and kneading was prolonged up to 15 min.

The resulting dough was divided into 8 aliquots, 150 g each, placed into baking molds, and leavened in a climatic chamber (Haereus Vötsch, mod. HC0020; Frommern, Germany) at 25 °C and 80% of relative humidity for different proofing times, depending on the leavening agent used (SD-doughs, 3 h; CY, 1 h 30 min; SDCY, 1 h). The optimal fermentation time, in fact, was defined for each dough by means of the rheofermentographic test (Section 2.2). The leavened dough was then baked in an electric oven (Lotus S.r.l., San Vendemiano, Treviso, Italy) for 30 min at 230 °C (bottom)–200 °C (top), then cooled at room temperature for 1 h, and finally removed from the molds.

### 2.4. Gluten-Free Bread Characterization

Bread evaluations were performed on two loaves for each GF bread recipe, at each sampling time: 1 h after baking (t0), and 23 h (t1), 46 h (t2), and 69 h (t3) after baking. During the experimental period, loaves were packed in paper bags and stored under controlled conditions (25 °C, 60% of relative humidity).

Fresh breads were characterized for weight (g; *n* = 8), maximum and minimum height (cm; calliper; 2 replicates; *n* = 16), volume (mL; AACC Method 10-05.01, replacing rapeseeds with sesame seeds; *n* = 8), and specific volume (mL/g; *n* = 8). 

At each sampling time, two bread loaves per recipe were weighed (to follow weight losses during storage time; %), sliced, and further characterized. The two back ends of each loaf were used for the evaluation of bread crumb porosity (by means of Image Analysis) and for color determination, while the three central slices (20-mm-thick) were used for the evaluation of bread moisture, water activity, and softness. 

The moisture of the central slice (*n* = 2), and of the crumb core (*n* = 4), was determined according to the AACC Method 44-15A (2000). The crumb core water activity (aw; *n* = 2) was measured by the Octagon Aqualab Series 3 (Decagon Devices Inc., Pullman, WA, USA).

Bread crumb softness was investigated through a TA-HD*plus* Texture Analyzer (Stable Micro Systems, Godalming, Surrey, UK) using a Texture Profile Analysis (TPA) test (500 N load cell). From each slice, a cylindrical crumb sample (25 mm diameter, 20 mm height) was obtained, and then compressed up to 25% deformation, using a 100 mm diameter cylindrical probe moving at a compression speed of 2 mm/s. From the resulting stress versus strain curves, the crumb hardness (N; load at 25% deformation) and the Young’s modulus (N/mm^2^; slope of the initial linear trait of the compression curve) were considered. At least 6 replicates (*n* ≥ 6) were performed for each bread recipe at each storage time. 

Bread crumb porosity was investigated via Image Analysis. The images of the two back ends of each bread loaf (*n* = 16) were acquired at 600 dpi with a HP ScanJet 8300 (Hewlett-Packard Development Company, Palo Alto, CA, USA), and processed by means of the software Image Pro-Plus (v. 4.5.1.29; Media Cybernetics, Rockville, MD, USA). A portion of each slice (always of the same size of 721 mm^2^) was selected and analyzed. The objects (holes) were counted and classified into 3 groups on the basis of their size: 0.1 < *x* ≤ 1 mm^2^; 1 < *x* ≤ 3 mm^2^; and *x* > 3 mm^2^. The following parameters were considered for each class: holes distribution (percentage of the total mean number of counted holes; %), holes area (percentage of the total alveolate area; %), and holes mean diameter (mm); furthermore, the total mean alveolate area (total holes area in the portion of the crumb analyzed; %) was quantified. 

Bread crust (*n* = 8) and crumb (the two back ends of each bread loaf; *n* = 16) color were measured using a Minolta Chroma Meter CR 210 (Minolta, Osaka, Japan). Results were expressed in the CIELAB color space, as L* (lightness; 0 = black, 100 = white), a* (+a = redness, −a = greenness), and b* (+b = yellowness, −b = blueness).

### 2.5. Statistical Analysis

Data were processed with STATGRAPHIC^®^ Plus for Windows 5.1 (StatPoint, Inc., Herndon, VA, USA), performing a one-way analysis of variance (ANOVA). Fisher’s least significant differences test was used to describe means at a 5% significance level.

## 3. Results and Discussion

### 3.1. Gluten-Free Dough Properties

*Dough consistency (water addition).* Dough consistency is a crucial parameter in breadmaking. Generally, with regard to wheat bread doughs, a 500 BU consistency is considered as “optimal” for the rheological properties of doughs and the final quality of breads. On the contrary, with regard to GF breads, such specific and robust indications are not yet available, and various consistency levels can be found in the scientific literature to produce an acceptable GF dough, essentially as a function of the GF recipe adopted. When using the same GF recipe, a lower consistency (i.e., higher water levels in the dough) has been found as preferable to assure good dough performances during leavening, in particular when ingredients having a high water affinity are included into the recipe [33]. A recent review revealed that textural parameters of GF crumbs are strongly related to dough consistency, and that a low dough consistency corresponds to a softer crumb [30]. The use of low GF bread dough consistency was also adopted in a recent study on the use of high pressure treated raw materials in GF baking [32]. However, the consistency cannot be too low. Finding a proper balance is therefore the crucial point in GF.

In some previous studies [27,31], the authors investigated a 230 BU GF bread dough consistency. In this research, 180 ± 10 BU was chosen as a reference for all the reasons stated above. The sample containing sourdough, which is usually the most critical in terms of leavening properties and dough development, was replicated at a 230 BU level consistency for comparison. The amounts of water required to reach the desired consistencies were the following (expressed as water absorption; WA, %): SD230, 57.8%; SD180, 64.5%; CY180, 82.0%; SDCY180, 64.5%. As already observed in the previous study [27], being SD and SDCY recipes with 20% of a high moisture ingredient, they required less water to reach the desired consistency. 

**Figure 1:**
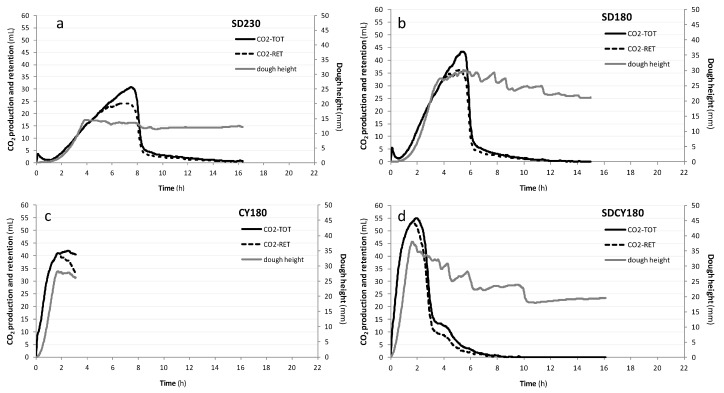
Rheofermentographic profiles of the different doughs: (**a**), dough obtained by adding 20% SD to the (gluten-free) GF bread recipe and having a farinographic consistency of 230 ± 10 BU (Brabender Unit); (**b**), dough obtained by adding 20% SD to the GF bread recipe and having a farinographic consistency of 180 ± 10 BU; (**c**) dough obtained by adding 2% of fresh compressed yeast to the GF bread recipe and having a farinographic consistency of 180 ± 10 BU; (**d**) dough obtained by adding 20% SD and 2% CY to the GF bread recipe and having a farinographic consistency of 180 ± 10 BU.

*Dough leavening properties*. The development of the various doughs during leavening, as well as their CO_2_ production and retention, as measured by the rheofermentographic test, were used to set up the proofing times to be adopted for each formulation during the real breadmaking process. Therefore, based on the previous experience [27], the test was carried out for different lengths in relation to the leavening agent used (SD230, 16 h 30 min; SD180, 15 h; CY180, 3 h; SDCY180, 16 h). In particular, for those doughs containing the SD, the test was stopped when a pH of 4.0 ± 0.2 was reached. The rheofermentographic profiles of the various doughs are reported in Figure 1. 

As expected, SD-doughs required longer periods of time to actively start the proofing, in comparison to the other dough systems. However, after that lag-period, SD microorganisms started their fermentation process, as well. In particular, the higher presence of water in SD180 probably guided the quicker and more intense height development of the dough, with respect to SD230 (Hm = 30.1 mm, SD180 vs. Hm = 14.5 mm, SD230). The higher amount of water in the dough could have been, at the same time, the cause of the larger decrease in SD180 dough height at the end of the test in comparison to SD230 (Hf = 21.1 mm, SD230 vs. Hf = 12.1 mm, SD180). Interesting dough height developments were reached in a very short period of time for CY180 (Hm = 28.2 mm; Hf = 26.2 mm), as expected. The recipe SDCY180 turned out to be very effective: the microorganisms, in fact, began the fermentation in a very short time and with a rapid growth (Hm = 38.1 mm), suggesting a synergistic effect among those microbial populations. To test this hypothesis, the development rates of these last three samples (SD180, CY180, and SDCY180) were quantified via linear interpolation (*R* > 0.997) of the development curves between 5 and 20 mm of dough height. The following dough height development kinetics were obtained: SD180, 13.33 mm/h; CY180, 24.13 mm/h; SDCY180, 29.70 mm/h. The positive effect of combining SD and CY in the same system is thus clear. Even if their proofing rates were largely different, the maximum dough height developments were similar for all of these samples, suggesting that all of the leavening agents taken into consideration were suitable for the processing conditions adopted in this study. 

Dough heights are generally a reflection of CO_2_ production and retention in the system. As can be appreciated from Figure 1, dough heights generally increase as long as the doughs are able to completely retain inside all of the produced CO_2_. As soon as Tx appears (time when the dough begins to give off CO_2_), a stability or a decrease in dough height is attained, in relation to the system’s strength [1]. Tx was equal to 5 h, 3 h 43 min, 1 h 52 min, and 1 h 43 min for SD230, SD180, CY180, and SDCV180, respectively. All the observations previously reported are congruent with these findings. 

As regards the doughs retention coefficients (Rc, %), the highest value was observed for CY180 (Rc = 95.2%), while the others were more close and similar to each other (Rc = 87.6%, SD230; Rc = 89.0%, SD180; Rc = 88.3%, SDCY180). However, when considering data related to CO_2_ production and retention, the influence of the leavening agent, the water level, the dough’s strength, and the test duration should be taken into account contemporarily.

On the basis of the rheofermentographic results, and particularly considering the Tx values, the following leavening times for bread production were defined: SD230, 3 h; SD180, 3 h; CY180, 1 h 30 min; SDCY180, 1 h. These times were slightly shorter than their corresponding Tx, since during the first period of baking, a further dough volume increase generally takes place. GF dough systems are usually very weak, and prolonging the proofing phase too much could result in a collapse (instead of a further development) of the structure during baking. 

*Dough pH evolution during proofing*. In parallel to the rheofermentographic test, pH evolution during proofing was recorded; for those doughs containing the SD, the test was stopped when a pH of 4.0 ± 0.2 was reached. The pH values of the various samples at the beginning of the rheofermentographic test were the following: SD230, 5.28; SD180, 5.30; CY180, 5.51; SDCY180, 5.13. During the test, pH decreased (curves not reported) by 1.35 and 1.36 units for SD230 and SD180, of 0.51 for CY180 and of 1.02 for SDCY180. 

### 3.2. Gluten-Free Bread Properties

*Features of the fresh products*. The main characteristics of the GF fresh breads 1 h after baking are reported in Table 2. Significant differences (*p* < 0.05) were evidenced among the samples after baking: loaves weights varied at 123.1 ± 1.3 g (SDCY180-bread) and 135.0 ± 1.0 g (SD180-bread), and baking losses were equal to 13.0% for SD230-bread, 10.0% for SD180-bread, 10.1% for CY180-bread, and 17.9% for SDCY180-bread. 

**Figure 2:**
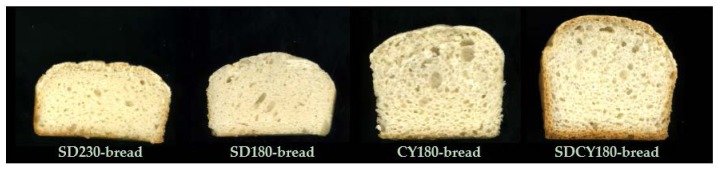
Cross sections of the various gluten-free (GF) loaves showing crumb structure.

Significant differences (*p* < 0.05) were also evidenced in terms of maximum height, minimum height, volume, and specific volume. For all these parameters, samples could be ordered as follows (from the lowest to the highest value): SD230-bread < SD180-bread < CY180-bread < SDCY180-bread. The best performances, therefore, were reached when a 180 BU dough consistency was adopted. More specifically, SDCY180-bread exhibited the highest values in terms of geometrical features (Table 2; Figure 2). As reported in other studies, the addition of sourdough to GF bread containing yeast as a leavening agent generally does not exert a remarkable influence on its specific volume [7,34,35]. On the contrary, in the present study, the contemporary presence of CY and SD determined a 19.5% increase in bread specific volume (with respect to CY180-bread specific volume), suggesting that the metabolites produced by the lactic acid bacteria were effective in modifying the rheological properties of SDCY180 dough, improving its deformation capability during proofing and baking. 

Another important parameter for consumer acceptability is bread softness, which is related to both the crumb porosity and the characteristics of the alveolar walls. GF bread texture was evaluated by a compression test during which the resistance offered by the crumb to the compression was continuously recorded. Young’s modulus was calculated from the resulting stress versus strain curve: the higher the value, the higher the loaf hardness. The two SD-loaves were significantly different from the other samples, and between them: as expected, the SD230-loaf exhibited the highest Young’s Modulus. As can be determined from Figure 2, these bread loaves were characterized by a denser and heavier crumb structure, in comparison to the others. The two breads containing CY, on the contrary, were much softer and not significantly different between them, independently of the amount of water required to reach the 180BU dough consistency (82.0% for CY180; 64.5% for SDCY180) and from their slice and crumb moisture values (Table 2).

These results were aligned with the crumb porosity features (Table 3; Figure 2): SD230-bread and SD180-bread crumbs were very dense, with a high percentage of holes (>90%) and a mean diameter of 0.6 mm, whereas CY180-bread and SDCY180-bread crumbs also exhibited a high amount (17%) of pores with an intermediate size (1 < *x* ≤ 3 mm). The presence of big holes (mean diameter > 2.2 mm) was very restricted in all the samples, indicating a limited structure breakdown during leavening and baking. Overall, SD230-bread and SD180-bread were characterized by a minor total mean alveolate area (14.0% and 16.5%, respectively), whereas CY/SD180-bread and CY180-bread showed values of 24.8% and 21.0%, respectively. All of these features are critical points in building up the final GF breads quality. As reported in [27], variations in bread crumb cellular structures (in addition to moisture variations)—which are themselves strictly dependent from formulation and processing conditions—are key contributors to changes in the final bread eating quality.

**Figure 3:**
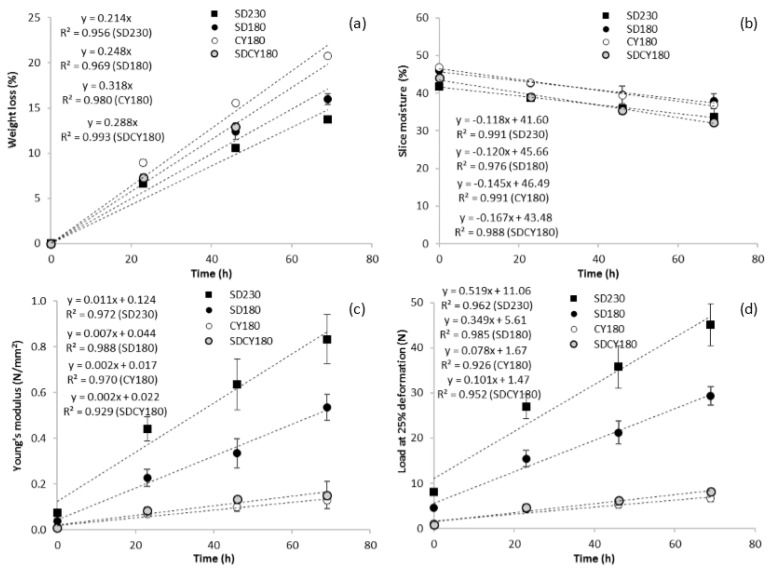
GF breads features evolution during storage (25 °C, 60% of relative humidity, in paper bags): (**a**) weight loss; (**b**) slice moisture; (**c**) crumb hardness: Young’s modulus; (**d**) crumb hardness: load at 25% deformation.

*Features of the products during storage*. Samples were also monitored under fixed times (23 h, 46 h, and 69 h) during storage under controlled conditions (Figure 3). Breads were packed in hand-folded paper bags to mimic a domestic shelf life and to enhance samples performances. 

Breads weight losses were more limited for SD230-bread and SD180-bread, in comparison to those containing CY (Figure 3a), whereas the reductions in slice moisture values were quite evident (18%–27%) for all the samples (Figure 3b).

As already evidenced for the fresh products, S230-bread and SD180-bread were also characterized during the whole storage period by the highest values of Young’s modulus and load at 25% deformation, and—in contrast with the expectations—by faster hardening kinetics as well (Figure 3c,d). These results did not seem to be directly related to the moisture levels of the various products; on the contrary, bread crumb structure (denser and heavier for SD230- and SD180-breads) seemed to be the main cause. At the opposite, CY180-bread and SDCY-bread were characterized by the lowest (and very close) values of Young’s modulus and load at 25% deformation during the whole storage period, as well as by the slowest hardening kinetics (Figure 3c,d). However, CY180-bread exhibited a crumbly behavior just after 30 h of storage. This undesirable characteristic could be related to the lower tendency of CY180-bread to bind water during storage, also evidenced by its weight losses (Figure 3a), thus confirming the findings of the previous work [27]. In that study, in fact, what was thought to be “softness” was on the contrary a higher “fracturability” of the sample, which indicated a higher tendency to staling. However, when the two leavening agents were combined in the SDCY180-bread, a synergistic effect was observed, and the resulting bread—despite its intermediate weight losses—did not show a crumbly behavior. In this case, therefore, the softness was a “real” softness and not a consequence of a higher fracturability of the crumb. 

## 4. Conclusions

The positive effects of sourdough on traditional breadmaking have been demonstrated by numerous studies. The results of this work support the assumption that a GF sourdough is a possible strategy to improve the quality of GF breads. However, the use of an in-lab-developed Type I GF sourdough (20% of the recipe) as the only leavening agent generated GF breads characterized by low developments and rapid staling. On the contrary, the contemporary presence of the two leavening agents (SD and CY) resulted in well-developed end products, characterized by a softer crumb and more reduced staling kinetics. A shorter fermenting period was required, in comparison to the other recipes, and the positive impact of the combination of the two leavening agents was also verified in terms of crumb structure and shelf-life extension of the end product. As regards the other process variable investigated in this study, a lower consistency (180 BU instead of 230 BU) demonstrated superior performance in terms of dough workability and end product quality. However, future research directed to investigate the SDCY-bread shelf life over a longer period, perhaps combining different packaging solutions, is necessary to better define the performance characteristics of this developed sourdough. 

## Figures and Tables

**Table 1:** Recipe of the various gluten-free doughs, water excluded.

Ingredient (%)	SD230	SD180	CY180	SDCY180
Corn starch	31	31	38	30
Rice flour	31	31	38	30
Isolated pea protein	5	5	6	5
Psyllium fiber	1.2	1.2	1.5	1.2
Hydroxyl propyl methylcellulose	1.2	1.2	1.5	1.2
Emulsifiers	0.4	0.4	0.5	0.4
Oil	5.0	5.0	6.1	4.9
Maltose	3.3	3.3	4.1	3.2
NaCl	1.7	1.7	2.0	1.6
SD	20	20	0	20
CY	0	0	2	2

**Table 2:** Gluten-free breads: Main features of the fresh products.

	SD230	SD180	CY180	SDCY180
**Bread weight** (g)	130.4 ± 1.4 ^b^	135.0 ± 1.03 ^c^	134.9 ± 1.2 ^c^	123.1 ± 1.3 ^a^
**Height max** (cm)	3.9 ± 0.1 ^a^	4.7 ± 0.2 ^b^	5.6 ± 0.1 ^c^	6.0 ± 0.2 ^d^
**Height min** (cm)	2.3 ± 0.10 ^a^	2.7 ± 0.1 ^b^	4.0 ± 0.2 ^c^	4.3 ± 0.3 ^d^
**Volume** (mL)	231.9 ± 9.5 ^a^	255.4 ± 6.2 ^b^	347.0 ± 8.4 ^c^	376.0 ± 9.1 ^d^
**Specific volume** (mL/g)	1.78 ± 0.07 ^a^	1.89 ± 0.05 ^b^	2.57 ± 0.08 ^c^	3.07 ± 0.09 ^d^
L*-crust	79.2 ± 3.2 ^b^	82.2 ± 2.3 ^c^	81.3 ± 1.5 ^c^	77.3 ± 2.4 ^a^
a*-crust	0.8 ± 0.9 ^c^	−1.5 ± 0.2 ^b^	−1.9 ± 0.2 ^a^	1.1 ± 0.7 ^c^
b*-crust	20.0 ± 2.9 ^b^	16.9 ± 2.8 ^a^	17.0 ± 2.4 ^a^	20.4 ± 1.4 ^b^
L*-crumb	74.3 ± 2.0 ^b^	75.1 ± 1.1 ^c^	72.6 ± 1.1 ^a^	74.3 ± 0.9 ^b^
a*-crumb	−2.06 ± 0.3 ^bc^	−1.96 ± 0.2 ^c^	−2.16 ± 0.2 ^ab^	−2.29 ± 0.2 ^a^
b*-crumb	16.4 ± 0.4 ^c^	14.9 ± 0.3 ^b^	12.6 ± 0.9 ^a^	12.8 ± 0.6 ^a^
**Water activity**	0.980 ± 0.001 ^a^	0.992 ± 0.001 ^c^	0.997 ± 0.001 ^d^	0.989 ± 0.001 ^b^
**Slice moisture** (%)	41.86 ± 0.14 ^a^	46.06 ± 0.02 ^c^	46.85 ± 0.14 ^d^	43.99 ± 0.25 ^b^
**Crumb moisture** (%)	47.98 ± 0.55 ^a^	50.40 ± 0.04 ^b^	51.05 ± 0.42 ^b^	53.01 ± 0.36 ^c^
**Young’s modulus** (N/mm^2^)	0.073 ± 0.002 ^c^	0.038 ± 0.003 ^b^	0.009 ± 0.001 ^a^	0.007 ± 0.002 ^a^
**Load 25% deformation** (N)	8.16 ± 0.073 ^c^	4.52 ± 0.28 ^b^	1.06 ± 0.05 ^a^	0.84 ± 0.12 ^a^

Note: Values followed by different letters in the same raw are significantly different (*p* < 0.05).

**Table 3:** Gluten-free breads crumb porosity features.

Sample	Holes Features			
	Size (mm^2^)	Distribution (%)	Area (%)	Diameter (mm)
**SD230-bread**	0.1 < *x* ≤ 1	90.2 ± 1.6 ^b^	62.50 ± 4.5 ^b^	0.6 ± 0.1 ^b^
	1 < *x* ≤ 3	8.8 ± 1.8 ^b^	27.63 ± 5.2 ^b^	1.4 ± 0.1 ^ab^
	*x* > 3	1.1 ± 0.6 ^a^	9.86 ± 5.5 ^a^	2.2 ± 0.6 ^a^
**SD180-bread**	0.1 < *x* ≤ 1	94.0 ± 1.5 ^c^	67.9 ± 8.6 ^c^	0.6 ± 0.1 ^a^
	1 < *x* ≤ 3	5.3 ± 1.5 ^a^	20.2 ± 5.1 ^a^	1.4 ± 0.1 ^ab^
	*x* > 3	0.7 ± 0.3 ^a^	13.0 ± 10.4 ^ab^	2.7 ± 0.7 ^b^
**CY180-bread**	0.1 < *x* ≤ 1	80.7 ± 3.6 ^a^	44.5 ± 6.6 ^a^	0.6 ± 0.1 ^c^
	1 < *x* ≤ 3	16.8 ± 3.0 ^c^	38.0 ± 5.9 ^c^	1.4 ± 0.1 ^b^
	*x* > 3	2.5 ± 1.1 ^b^	17.5 ± 8.6 ^b^	2.3 ± 0.2 ^a^
**SDCY180-bread**	0.1 < *x* ≤ 1	80.8 ± 3.5 ^a^	47.0 ± 6.4 ^a^	0.7 ± 0.1 ^d^
	1 < *x* ≤ 3	17.1 ± 3.3 ^c^	37.8 ± 6.1 ^c^	1.4 ± 0.1 ^a^
	*x* > 3	2.1 ± 0.8 ^b^	15.2 ± 7.7 ^ab^	2.3 ± 0.2 ^ab^

Note: Within the same holes size class, values followed by different letters in the same column are significantly different (*p* < 0.05).
